# Goat uterine epithelial cells are susceptible to infection with Caprine Arthritis Encephalitis Virus (CAEV) in vivo

**DOI:** 10.1186/1297-9716-43-5

**Published:** 2012-01-25

**Authors:** Mohamad Z Ali Al Ahmad, Laurence Dubreil, Gérard Chatagnon, Zakaria Khayli, Marine Theret, Lionel Martignat, Yahia Chebloune, Francis Fieni

**Affiliations:** 1 LUNAM University, Oniris, Nantes-Atlantic National College of Veterinary Medicine, Food Science and Engineering, Sanitary Security of Reproduction Biotechnology Unit, F-44307 Nantes, France; 2 Department of Surgery and Obstetrics, Faculty of Veterinary Medicine - University of Al-Baath, Hama, Syria; 3 INRA, UMR 703, F-44307 Nantes, France; 4 LUNAM University, Oniris, Nantes-Atlantic National College of Veterinary Medicine, Food Science and Engineering, Muscle Tissue Development and Pathology Unit, F-44307 Nantes, France; 5 LAPM, UMR 5163, CNRS 38042 Grenoble, France; 6 CNPRC Infectious Diseases Unit, Center of comparative medicine, UC DAVIS CA, USA

## Abstract

The aim of this study was to determine, using immunofluorescence and in situ hybridization, whether CAEV is capable of infecting goat uterine epithelial cells in vivo. Five CAEV seropositive goats confirmed as infected using double nested polymerase chain reaction (dnPCR) on leucocytes and on vaginal secretions were used as CAEV positive goats. Five CAEV-free goats were used as controls. Samples from the uterine horn were prepared for dnPCR, in situ hybridization, and immunofluorescence. The results from dnPCR confirmed the presence of CAEV proviral DNA in the uterine horn samples of infected goats whereas no CAEV proviral DNA was detected in samples taken from the uninfected control goats. The in situ hybridization probe was complementary to part of the CAEV *gag *gene and confirmed the presence of CAEV nucleic acids in uterine samples. The positively staining cells were seen concentrated in the mucosa of the lamina propria of uterine sections. Finally, laser confocal analysis of double p28/cytokeratin immunolabelled transverse sections of CAEV infected goat uterus, demonstrated that the virus was localized in glandular and epithelial cells. This study clearly demonstrates that goat uterine epithelial cells are susceptible to CAEV infection in vivo. This finding could help to further our understanding of the epidemiology of CAEV, and in particular the possibility of vertical transmission.

## Introduction

Caprine arthritis-encephalitis virus (CAEV) was first described as a cause of chronic arthritis in American goats [1-3], and has since been found to be widespread in goat herds worldwide [4,5]. CAEV is an RNA virus belonging to the *lentivirus *genus of the family *retroviridae *[1]. In France, the infection is present in around 80 to 95% of breeding herds [6] and causes economic losses through reduced milk production, early culling, and loss of export potential [7]. Symptoms of infection may include lung disease and, more often, indurative mastitis as well as classical arthritis. Leucoencephalitis in young kids [1] remains rare.

Infection can be transmitted by any means involving the transfer of infected cells to a naïve recipient. In the field, the principal route of transmission is vertical from dam to kids through colostrum and milk [5], with additional horizontal transmission following prolonged contact between infected and naïve adult animals [8]. Attempts to reduce infection by treating colostrum and milk, and separating infected and naïve animals have been disappointing [8,9], and attempts have been made to identify other risk factors [10,11].

Although the oral route remains the principal mode of natural transmission, sexual transmission has yet to be fully explored. CAEV proviral DNA has been identified using PCR in tissues of the genital tract (uterus, oviduct, and ovary [12]), and in uterine flushing media recovered four days after fecundation [13,14].

The virus primarily infects cells of the monocyte-macrophage lineage, with viral production being linked to cell differentiation from monocytes to macrophages [15,16]; however, viral transcripts have been detected in epithelial cells in the small intestine, thyroid gland, and kidneys of infected goats [17]. In vitro, granulosa cells, oviduct epithelial cells [18,19] and caprine early embryonic cells [20] are susceptible to CAEV infection, and infection with this virus is productive. Nevertheless, no information is available concerning the phenotype of CAEV infected cells in the female genital tract. This information would improve our understanding of the risk of CAEV vertical transmission in utero or following in vivo as well as in vitro embryo production and embryo transfer.

The goal of this study was to determine, using immunofluorescence and in situ hybridization, whether CAEV is capable of infecting uterine epithelial cells in vivo.

## Materials and methods

### Animals

Five goats that had repeatedly tested seropositive for CAEV using ELISA, and that were confirmed as positive using PCR on leucocytes and vaginal secretions, were used as positive infected goats. Five goats that were selected from ELISA certified CAEV negative herds and which had two dnPCR negative blood samples and two dnPCR vaginal swab samples at an interval of one month were used as negative control goats.

### Samples

The goats were slaughtered in accordance with French regulations. Immediately prior to slaughter, 8 mL of blood were drawn from the jugular vein into acid citrate dextrose.

The uterus was harvested from each animal immediately after euthanasia and exsanguination. Uterine samples were taken from each goat from the greater curvature of the uterine horn and processed in one of three different ways: for PCR analysis, samples were stored at -80°C. For immunohistochemistry analyses, samples were embedded in Tissue tek (Sakura Finetek, Torrance, CA, USA) and frozen in isopentane cooled with liquid nitrogen. Transverse cryosections of uterine horn were prepared with a Leica CM 3050S cryostat (Leica Microsystems, Nanterre, France). For in situ hybridization, tissues were fixed in 4% formaldehyde for 10 min at room temperature and embedded in paraffin. We used separate scalpel blades for each goat. When collecting different samples from the same goat, we washed the scalpel blade in RBS Viro (Fluka Chemical Corp New York NY, USA), wiped it on a sheet of absorbent paper, disinfected it with ethanol (70°), and finally dried it with a new sheet of absorbent paper between each sample.

### PCR analyses

#### Preparation of the samples for PCR

The whole blood, collected in anticoagulant, was centrifuged at 1900 × *g *for 30 min at room temperature using Ficoll density-gradient centrifugation. At the end of this first phase, leucocytes were recovered from the buffy coat, washed in sterile PBS pH 7.2, and centrifuged for 5 min at 700 × *g*, three times over. The supernatant was discarded and the cell pellet was stored at -80°C awaiting subsequent DNA extraction.

After thawing, DNA was extracted from the leucocytes and uterine samples using a "QIAamp Tissue kit^®^" (Qiagen, Courtabœuf, France), in accordance with the manufacturer's instructions. The samples were then stored at -20°C, awaiting PCR analysis.

#### Procedure for double nested-PCR

We used the double nested-PCR technique, as described previously by Barlough et al.[21] to detect CAEV proviral-DNA in the blood and uterine horn tissue samples. Two rounds of PCR amplification were used to detect the gag sequence of the CAEV genome.

In the first round, the virus was detected by amplifying a fragment of proviral-DNA, located between nucleotide 393 and nucleotide 1291, using external primers GAG EX5 (5'- GAA GTG TTG CTG CGA GAG GTC TTG -3') and GAG EX3 (5'- TGC CTG ATC CAT GTT AGC TTG TGC -3'). This round was immediately followed by a second round, amplifying the fragment located between nucleotide 524 and nucleotide 1036, using internal primers GAG IN5 (5'- GAT AGA GAC ATG GCG AGG CAA GT -3') and GAG IN3 (5'- GAG GCC ATG CTG CAT TGC TAC TGT -3'). Oligonucleotide primers specific to the fourth exon of the human β-actin gene were used as an internal control for the integrity of the DNA lysates: external-ES30 (5'- TCA TGT TTG AGA CCT TCA ACA CCC CAG -3') and ES32 (5'- CCA GGG GAA GGC TTG AAG AGT GCC -3') for the first round, and internal-ES31 (5'- CCC CAG CCA TGT ACG TTG CTA TCC -3') and ES33 (5'- GCC TCA GGG CAG CGG AAC CGC TCA -3') for the second round [22].

For the first round of amplification, 10 μL of DNA (containing 0.5 to 1 μg) were added to 40 μL of an amplification solution, or "mix1". The latter was comprised of 5 μL of reaction buffer [10×] (670 mM Tris/HCl (pH 8.8), 160 mM (NH_4_)_2_SO_4_,0.1% TWEEN-20), 5 μL of MgCl_2 _(50 mM), 1 μL of dNTP (25 mM of each oligonucleotide triphosphate: dATP, dGTP, dCTP, dTTP), 0.25 μL of TAQ Polymerase (5 U/μL, EUROBIOTAQ^® ^DNA Polymerase-Thermostable, GAETAQ02K, EUROBIO, Les Ulis, France), 0.5 μL each of primers GAG EX3, GAG EX5, ES30, and ES32 (20 μM, GIBCO BRL Custom primers - Life Technologies, Grand Island, NY, USA), and 26.75 μL of DEPC-treated water.

For the second round, 5 μL of the first round were added to 45 μL of a second amplification solution, or "mix2" containing the same reagents as the solution in mixture 1, except that internal primers GAG IN5, GAG IN3, ES31, and ES33 were used in place of external primers GAG EX5, GAG EX3, ES30, and ES32. For each round, following initial denaturation at 94°C for 5 min, the samples were submitted to a series of 35 cycles comprising, successively, a further one-minute denaturation phase at 94°C, a 90-second hybridization phase at 46°C and a 2-and-a-half-minute extension phase at 60°C. Each round was followed by a final extension at 60°C for 15 min. Amplification products were visualized using electrophoresis on 1.5% agarose gel (GIBCO LIFE Technologies, Grand Island, NY, USA), containing Ethidium Bromide in 1× TAE buffer: 10 μL of the amplified fraction were added to 5 μL of dyed loading buffer, in each gel well. Two controls were performed for each gel: a positive control (CAEV proviral-DNA from infected GSM (goat synovial membrane)) and a negative control (distilled water). Five microliters of Smart-Ladder (GIBCO LIFE Technologies), was used as a molecular weight marker. This marker comprises 14 bands calibrated between 10 000 and 200 bp. Following electrophoretic separation, the bands were visualized using transillumination, with ultraviolet light (312 nm).

### In situ hybridization and cytokeratin labeling

#### In situ hybridization

In situ hybridization was used to detect the presence of CAEV nucleic acids in the uterine samples. The specificity of the probe was checked by the observation of in situ hybridization on two goat synovial membrane cell cultures infected in vitro with CAEV-3112 [23]. Mammary lymph node samples from PCR-confirmed CAEV-infected goats with clinical disease were used as positive controls. Negative controls included PCR-negative tissues (lymph node and uterus) and non-infected goat synovial membrane cell cultures. The absence of non-specific labeling was checked by omitting the labeled probe in serial sections of positive tissues (lymph node and uterus) and in goat synovial membrane cell cultures infected in vitro.

All tissues were paraffin embedded after formalin fixation, and all solutions used were made with di-ethyl-pyro-carbonate-treated water to inactivate eventual RNAse contamination. Tissue sections (ca 4 μm) were spread out on RNAse-free silanized slides [24], covered with PBS pH 7.6 and dried in a hood oven at 37°C for 12 h before being deparaffinized twice for ten minutes in 1-bromopropane (LMR-Ground) and re-hydrated through 100%, 95%, 70% and 30% ethanol baths (5 min each) and distilled water (30 s). After 3 × 3 min washes in PBS, the sections were treated with an enhancing solution comprising 62.5 μL acetic anhydride in 25 mL of 0.1 M triethanolamine pH 8.0 for 10 min, washed in SSC 1× for 5 min, permeabilised in 0.2 M HCl for 10 min at room temperature, and finally washed in PBS for 5 min before hybridization.

A probe complementary to viral strand RNA was generated from the pBSCA plasmid carrying the complete CAEV genome by PCR amplification of the gag region using primers GAG EX 5' and GAG EX 3'. After purification with a GeneClean kit (Appligene, Illkirch, France), the probe was labeled by nick translation [25] using the Dig-Nick translation kit (Rock GmbH, Mannheim, Germany), and unincorporated nucleotides were removed by passage through Probe Quant T.M.G 50 columns (Amersham, Orsay, France). Labeled probes were verified by slot-blot determinations on Hybond™ N+ nylon membranes (Amersham, Pantin, France), and stored at -20°C until use.

Slides were pretreated with 30 μL hybridization mixture (50% formamide in 5× SSC containing 2% Casein, 0.1 N N-lauroyl sarcosine and 200 μg/mL sheared denatured salmon sperm) for 1-2 h at 42°C. The labeled probe was denatured by boiling for 10 min, then cooling on ice immediately before hybridization. The slides were overlaid with 30 μL of hybridizing mixture containing 16-32 ng of labeled probe and preheated to 42°C, covered with coverslips, and incubated for 16 h at 42°C in a moist atmosphere. The reaction was terminated by removing the coverslips and washing the slides in 2× SSC containing 0.1% SDS, twice for 5 min. After two further washes in a solution containing 1× SSC and 0.1% SDS for 15 min at 42°C and a solution containing 0.1 M malic acid and 0.3% Tween 20 for 1 min at room temperature, the slides were blocked with 10% casein in malic acid for 30 min at room temperature. They were then treated with alkaline phosphatase-labeled anti-dioxygenin (1/5000 in blocking solution) for a further 30 min at room temperature. After two washes in malic acid for 15 min at room temperature and rinsing in AP buffer (100 mM Tris HCl, 100 mM NaCl and 50 mM MgCl_2 _in water), freshly-prepared chromogenic substrate (5-bromo-4-chloro-3-indolylphosphate/Nitro blue tetrazolium) in AP buffer was added for 10-20 min at room temperature in the dark. After rinsing in distilled water, the slides were counterstained with Harris hematoxylin, rinsed in tap water, dehydrated in alcohol, air dried, and mounted in DAKO^® ^(Faramount, Trappes, France).

#### Cytokeratin labeling

To identify epithelial cells in uterine sections, adjacent sections were stained for cytokeratin. The sections were treated with trypsin (1 mg/mL) for 30 s at 37°C, washed three times in distilled water, blocked with 3% hydrogen peroxide for 10 min, washed in PBS pH 7.6 for 5 min, and then in normal goat serum (Dako; X.907; 20% in PBS) for 20 min. They were then incubated with a monoclonal anti-cytokeratin antibody (Dako; M 821: 1/500) in 2% BSA in PBS for 60 min in a humid chamber, washed three times in PBS, and incubated with biotinylated goat anti-mouse IgG (Dako; E.433; 1:300 in PBS) for 30 min at room temperature, then with streptavidin/peroxidase (Dako; P.397; 1:300 in PBS) for a further 30 min at room temperature. After the final wash, the slides were treated with chromogenic reagent (diaminobenzidine; Dako K.3485) for 15 min, counterstained with Harris hematoxylin, and mounted as above. Unrelated murine antibodies of the same Ig class were used as negative controls and oviductal epithelial cell cultures as positive controls.

### Immunofluorescence and laser scanning confocal analysis

To determine the cellular localization of CAEV in uterine samples, we tested our primary antibody, IgG1 anti-p28 (CAEP5A1, VMRD, Pullman, USA), on goat synovial membrane cell cultures (GSM) infected in vitro by CAEV-3112. GSM are very sensitive to CAEV and therefore constitute a good positive control. Negative controls included PCR-negative uterine tissues and non-infected GSM cell cultures. Furthermore, the absence of non-specific labeling was checked by omitting the primary antibody on positive uterine tissue. To pinpoint the cellular localization of virus in uterine tissues, we performed double labeling against p28 and cytokeratin (N75350, Interchim, Montluçon, France) as a marker of epithelial cells, in combination with nuclei staining.

Transverse cryosections of uterine horn (10 μm) were prepared using a Leica CM 3050S cryostat. Sections were air dried at room temperature and fixed for 15 min with acetone at 4°C before being stored at -80°C. Immunolabeling was performed on freshly thawed sections. Sections were rehydrated with PBS and blocked with 10% donkey serum diluted in PBS. The mouse anti-p28 monoclonal antibody was diluted to 1/100 in blocking buffer and sections were incubated with this primary antibody overnight at 4°C. After washing, the sections were incubated with the secondary antibody, Alexa Fluor 555 donkey anti-mouse, for one hour at room temperature (1/400, A31570, Invitrogen, France). After washing, the samples were then fixed with PFA 4% for 15 min at room temperature, washed in PBS and incubated with a mouse anti-cytokeratin monoclonal antibody (N75350, Interchim, Montluçon, France) overnight at 4°C. Sections were then washed in PBS and incubated with a secondary antibody, Alexa fluor 488 donkey anti-mouse (A21202, Invitrogen, France). Nuclei were stained with Topro-3 (1/1000, Invitrogen Life Technologies, Illkirch, France). Finally, slides were coverslipped and mounted with Mowiol Medium (Clabiochem EMD Biosciences, San Diego, CA, USA). The immunolabeled sections were serially scanned with a confocal microscope (Nikon C1, Champigny-sur-Marne, France) using the 543 nm helium neon laser to observe p28 immunolabeling (red), the argon ion laser (488 nm) to observe cytokeratin immunolabeling (green), and the 633 nm helium neon laser to observe stained nuclei (blue).

## Results

### PCR results

Samples analyzed for CAEV proviral-DNA using PCR were considered to be positive when a 512 bp band, corresponding to the positive control, was seen on agarose gel electrophoresis under UV light, between the 600 bp and 400 bp molecular weight bands. Whereas the 393 bp band, generated from the amplification of the endogenous actin gene, was present in both non-infected and CAEV-infected samples.

Nested-PCR amplification using DNA isolated from white blood cells and uterine samples taken from positive goats gave clear bands of the expected size (512 bp) on agar gel electrophoresis. No CAEV proviral DNA was detected in samples taken from non-infected control goats.

### In situ hybridization

**Figure 1:**
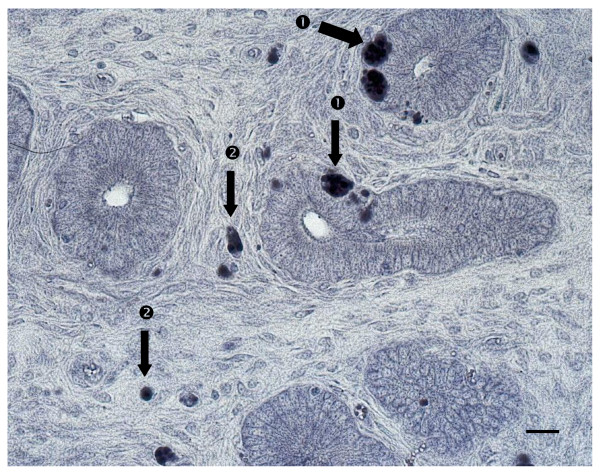
**In situ hybridization of uterine tissues (bar = 10 micrometers)**. CAEV RNA positive staining (brownish-purple intracytoplasmic inclusions) could be identified (black arrow) in the uterine glands ① and in the lamina propria of the mucosa ② of uterine sections.

**Figure 2:**
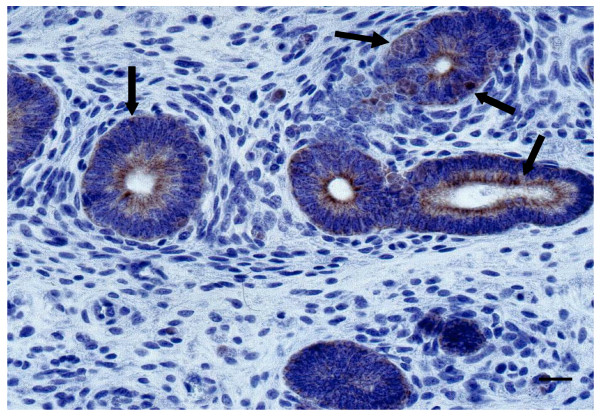
**Cytokeratine labeling of figure 1 adjacent section (4 μm)**. The brown cytokeratin staining (black arrow) identified the single layer of cuboidal glandular epithelial cell (bar = 10 micrometers).

Hybridizing cells were characterized by the presence of brownish-purple intracytoplasmic inclusions. The positively-staining cells were concentrated in the mucosa of the lamina propria of uterine sections (Figure 1) and the uterine glands. In the adjacent sections, the glandular epithelial cells could be distinguished by their cuboid morphology with nuclei at the lower end, and were confirmed by brown cytokeratin staining (Figure 2).

### Immunofluorescence

**Figure 3:**
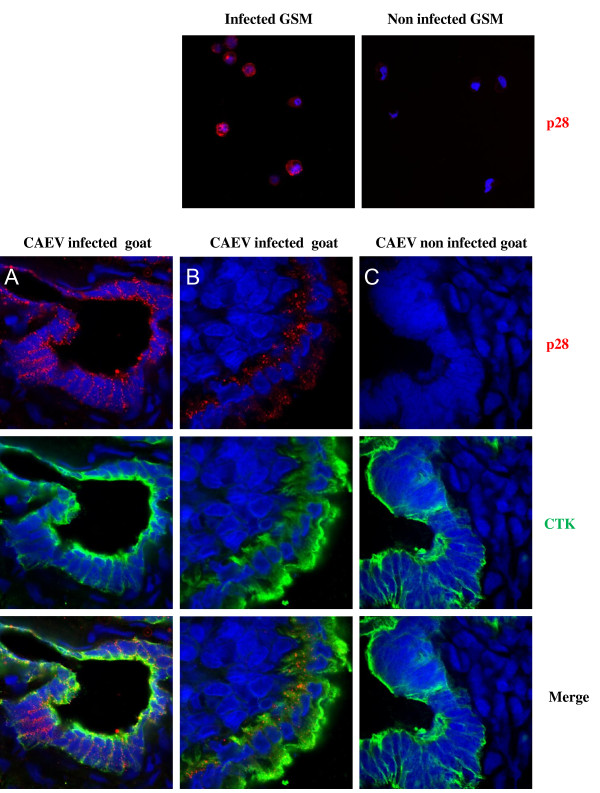
**Immunofluorescence results after laser scanning confocal analysis of (i) single CAEV p28 labeling (red color) on CAEV infected and non-infected GSM cells and of (ii) double CAEV p28 (red color) and cytokeratin (green color) labeling on the same uterine section of CAEV infected and non-infected goats**. A: uterine gland from a CAEV infected goat; B: external epithelium of a uterus from a CAEV infected goat; C: an external epithelium of a uterus from a non infected goat.

Immunohistochemical analysis of CAEV p28 was performed on infected and non-infected GSM. Specific labeling of the virus was observed on CAEV infected GSM whereas no uptake occurred in healthy GSM. The double p28/cytokeratin immunolabeling performed on transverse sections of CAEV-infected goat uterus revealed that the virus was essentially localized in glandular epithelial cells and in the external epithelial cells of the uterus. No uptake was seen in non-infected goat uterus sections (Figure 3).

## Discussion

In this study, we investigated the cellular localization of CAEV in uterine tissues taken from CAEV-infected goats, using in situ hybridization and immunofluorescence with laser scanning confocal analysis.

CAEV infection of uterine tissues was confirmed by the presence of cells with clear and specific cytoplasmic hybridization to a CAEV-specific probe. We used a probe that is a complementary part of the CAEV *gag *gene; a detectable reaction requires the presence of several copies of target sequences. The reaction thus revealed cytoplasmic concentrations consistent with actively replicating CAEV, and not just nuclear localizations of proviral sequences.

The double immunolabeling against p28 and cytokeratin, analyzed using immunofluorescence and laser scanning confocal analysis, clearly demonstrated that uterine epithelial cells were infected by CAEV in vivo.

In vitro studies have shown that various epithelial cell types from the goat genital tract, oviduct epithelial cells [18], and granulosa cells [19], are highly susceptible to productive infection with CAEV. In other species various lentiviruses, including Feline Immunodeficiency Virus (FIV) [26], Simian Immunodeficiency Virus (SIV) [27], and the Human Immunodeficiency Virus (HIV-1) [28] have been shown to infect cells of the epithelial lineage.

The presence of CAEV-infected epithelial cells in the genital tract tissues could have a major impact on the epidemiology of the disease. Infected epithelial cells could maintain latent infection with no accompanying inflammatory reaction; the embryo or fetus could therefore come into contact with CAEV during pregnancy and be infected at different stages of development. This would explain the presence of amplifiable CAEV sequences in flushing media from superovulated does [13,14] and in the post partum secretions of breeding goats [10]. Even if the significance of intrauterine viral transmission continues to be unclear and controversial [29], infected epithelial cells could also explain the seroconversion in 2 of 32 kids delivered by caesarean section, or 1 in 10 naturally born animals, all of which were colostrum-deprived [8]. In another instance, nearly a quarter of kids separated from their mothers at birth had seroconverted to CAEV by 5 months [30]. The related ovine lentivirus has been shown to infect fetal lambs in utero [31] and it has been estimated that this route could account for 11% of contamination of lambs [32].

The presence of the CAEV genome in the uterine epithelial cells of does raises concerns regarding the risk of CAEV transmission via embryo transfer. After in vitro fertilization, early stage embryos are cultured on a feeder layer of epithelial cells from goat oviducts [33-35], which are essential for early development before transfer to recipient does [36,37]. These cells are derived from organs obtained at the slaughterhouse from goats of unknown CAEV status, and in many industrialized countries the incidence of CAEV infection may be as high as 60 to 80% [6]. A similar instance occurred in bovine embryo biotechnology, where feeder cells were found to carry bovine viral diarrhea virus from contaminated donors [38], which eventually infected embryos [39]. The risk of embryo contamination by CAEV would seem, however, to be limited, provided that the guidelines of the International Embryo Transfer Society [40], are followed strictly, because an intact zona pellucida has been shown to protect against CAEV infection [14,41].

## Competing interests

The authors declare that they have no competing interests.

## Authors' contributions

FF and MZAAA conceived the study, participated in its design and coordination and wrote the manuscript. LD supervised the immunofluorescence and laser scanning confocal analysis. GC, ZK and MT carried out the sample collection, the PCR analysis and the immunofluorescence preparation and participated in the design. LM and YC carried out the in situ hybridation and the revision of the study. All authors read and approved the final manuscript.
